# Supplementary material for: Synthesis of light-inducible and light-independent anthocyanins regulated by specific genes in grape ‘Marselan’ (V. vinifera L.)
Source: PeerJ. 2019 Mar 1;7:e6521. doi: 10.7717/peerj.6521 (PMC6398381; doi:10.7717/peerj.6521)
Supplement: Supplemental Information 2 — Primer pairs used for qRT-PCR and RNA-Seq data [file peerj-07-6521-s002.docx]

**Table S1 Primer pairs used for qRT-PCR**

| **Gene ID** | **Gene name** | **Forward primer** | **Reverse primer** |
| --- | --- | --- | --- |
| VIT_06s0004g08150 | *C4H* | 5’-ACCACCTGAACCTCTCCGACTTAG-3’ | 5’-TTCTTGTGCGTGATCCGAACTCC-3’ |
| VIT_05s0136g00260 | *CHS* | 5’-CGACACGTCTTGAGCGAGTATGG-3’ | 5’-TCAGCCGACTTCCTCCTCATCTC-3’ |
| VIT_13s0019g04460 | *PAL* | 5’-CGGTGGAGCTGTCGGAGGAG-3’ | 5’-CGCCGTAGCTGTCTGTACCATTG-3’ |
| VIT_13s0067g03820 | *CHI* | 5’-TATTGACGGAGGCGGTACTGGAG-3’ | 5’-CAAGCCACCGGAGCAACCTTC-3’ |
| VIT_18s0001g14310 | *F3H* | 5’-AACCAGTTCAGCAACGAGATTCCG-3’ | 5’-CACACGCCTCCACAATCTTCCTAC-3’ |
| VIT_18s0001g12800 | *DFR* | 5’-CCTCCGCAGGAACTGTGAACATTC-3’ | 5’-GCAGCTTGCTCAGCCAGTGTC-3’ |
| VIT_02s0025g04720 | *LDOX* | 5’-CGGCAAGATCGCTGGCTATGG-3’ | 5’-CGTAGTCGCTTGGTGTCTTAGGC-3’ |
| VIT_06s0009g02970 | *F3'5'H* | 5’-TGGTCTCAGGTCCGAGCTGTTG-3’ | 5’-GCTCCACCACCATGTCCTTGAAC-3’ |
| XM_002266714 | *UBI* | 5’-GCTCGCTGTTTTGCAGTTCTAC-3’ | 5’-AACATAGGTGAGGCCGCACTT-3’ |

**Table S2 Summary raw data of transcriptome sequencing**

|  | **CK1** | **A1** | **B1** | **C1** | **D1** | **CK2** | **A2** | B2 | C2 | D2 |
| --- | --- | --- | --- | --- | --- | --- | --- | --- | --- | --- |
| Raw reads | 39760708 | 97000848 | 58600638 | 44545756 | 48564214 | 54697336 | 62782440 | 54073768 | 57071458 | 54386938 |
| Clean reads | 38872988 | 95514330 | 58126680 | 43956982 | 47650110 | 53592022 | 61878802 | 53313984 | 56324756 | 53430684 |
| GC Content | 47.42% | 49.42% | 47.16% | 47.46% | 46.80% | 47.53% | 47.29% | 47.28% | 47.66% | 47.08% |
| %≥Q30 | 90.45% | 90.47% | 92.90% | 91.02% | 90.92% | 90.35% | 90.71% | 90.69% | 90.82% | 90.65% |

**Table S3 Summary of transcriptome sequencing data**

|  | **CK1** | **A1** | **B1** | **C1** | **D1** | **CK2** | **A2** | **B2** | **C2** | **D2** |
| --- | --- | --- | --- | --- | --- | --- | --- | --- | --- | --- |
| Total reads | 38764792 | 95452038 | 57842426 | 43874400 | 47537330 | 53510908 | 61570652 | 53262246 | 55985036 | 53372358 |
| High-quality reads(%) | 97.77% | 98.47% | 99.19% | 98.68% | 98.12% | 97.98% | 98.56% | 98.59% | 98.69% | 98.24% |
| Mapped Reads(%) | 74.37% | 46.36% | 73.38% | 75.27% | 78.43% | 73.90% | 66.86% | 70.75% | 70.34% | 74.16% |
| Uniq Mapped Reads | 74.00% | 46.10% | 73.00% | 74.90% | 78.00% | 73.50% | 66.50% | 70.40% | 70.00% | 73.80% |
| Multiple Map Reads | 0.37% | 0.26% | 0.38% | 0.37% | 0.43% | 0.40% | 0.36% | 0.35% | 0.34% | 0.36% |

**Table S4 Gene Ontology (GO) enrichment analyses for DEGs between different treatments**

| **GO domain** | **GO terms ID** | **Gene Ontology term** | **Number of DEGs** | **Corrected *P*-value (<0.05)** |
| --- | --- | --- | --- | --- |
| **Biological_process** |  | **CK1 versus A1** |  |  |
|  | GO:0006950 | response to stress | 124/2023 | 0.001434 |
|  | GO:0019725 | cellular homeostasis | 19/225 | 0.010854 |
|  | GO:0042592 | homeostatic process | 19/226 | 0.011343 |
|  | GO:0065008 | regulation of biological quality | 19/226 | 0.011343 |
|  | GO:0050896 | response to stimulus | 174/3177 | 0.018193 |
|  | GO:0019748 | secondary metabolic process | 20/281 | 0.046360 |
|  |  | **CK1 versus B1** |  |  |
|  | GO:0009719 | response to endogenous stimulus | 95/618 | 0.000000 |
|  | GO:0050896 | response to stimulus | 348/3177 | 0.000007 |
|  | GO:0006950 | response to stress | 229/2023 | 0.000050 |
|  | GO:0009607 | response to biotic stimulus | 66/504 | 0.000964 |
|  | GO:0008219 | cell death | 28/186 | 0.004166 |
|  | GO:0007154 | cell communication | 142/1294 | 0.004783 |
|  | GO:0007165 | signal transduction | 118/1076 | 0.009878 |
|  | GO:0050794 | regulation of cellular process | 118/1077 | 0.009878 |
|  | GO:0051716 | cellular response to stimulus | 118/1077 | 0.010151 |
|  | GO:0009875 | pollen-pistil interaction | 17/107 | 0.013526 |
|  | GO:0023052 | signaling | 118/1106 | 0.021378 |
|  | GO:0044700 | single organism signaling | 118/1106 | 0.021378 |
|  | GO:0006464 | cellular protein modification process | 261/2658 | 0.041410 |
|  | GO:0036211 | protein modification process | 261/2658 | 0.041410 |
|  | GO:0043412 | macromolecule modification | 261/2658 | 0.041410 |
|  |  | **CK1 versus C1** |  |  |
|  | GO:0009607 | response to biotic stimulus | 55/504 | 0.000009 |
|  | GO:0009719 | response to endogenous stimulus | 63/618 | 0.000019 |
|  | GO:0008219 | cell death | 24/186 | 0.000280 |
|  | GO:0050896 | response to stimulus | 231/3177 | 0.000334 |
|  | GO:0007165 | signal transduction | 91/1076 | 0.000368 |
|  | GO:0050794 | regulation of cellular process | 91/1077 | 0.000380 |
|  | GO:0051716 | cellular response to stimulus | 91/1077 | 0.000380 |
|  | GO:0009605 | response to external stimulus | 61/671 | 0.000598 |
|  | GO:0023052 | signaling | 91/1106 | 0.000892 |
|  | GO:0044700 | single organism signaling | 91/1106 | 0.000892 |
|  | GO:0007154 | cell communication | 103/1294 | 0.001266 |
|  | GO:0009628 | response to abiotic stimulus | 82/1015 | 0.002631 |
|  | GO:0065007 | biological regulation | 113/1474 | 0.002668 |
|  | GO:0006950 | response to stress | 148/2023 | 0.003755 |
|  | GO:0044710 | single-organism metabolic process | 101/1367 | 0.012355 |
|  | GO:0015979 | photosynthesis | 27/288 | 0.012839 |
|  | GO:0050789 | regulation of biological process | 94/1264 | 0.013215 |
|  | GO:0019725 | cellular homeostasis | 22/225 | 0.014920 |
|  | GO:0042592 | homeostatic process | 22/226 | 0.015636 |
|  | GO:0065008 | regulation of biological quality | 22/226 | 0.015636 |
|  | GO:0006629 | lipid metabolic process | 84/1166 | 0.036461 |
|  | GO:0006810 | transport | 172/2577 | 0.049321 |
|  |  | **CK1 versus D1** |  |  |
|  | GO:0007165 | signal transduction | 208/1076 | 0.000015 |
|  | GO:0050794 | regulation of cellular process | 208/1077 | 0.000016 |
|  | GO:0051716 | cellular response to stimulus | 208/1077 | 0.000016 |
|  | GO:0023052 | signaling | 209/1106 | 0.000060 |
|  | GO:0044700 | single organism signaling | 209/1106 | 0.000060 |
|  | GO:0007154 | cell communication | 239/1294 | 0.000083 |
|  | GO:0050896 | response to stimulus | 537/3177 | 0.000112 |
|  | GO:0006091 | generation of precursor metabolites and energy | 84/414 | 0.001266 |
|  | GO:0065007 | biological regulation | 258/1474 | 0.001326 |
|  | GO:0009607 | response to biotic stimulus | 98/504 | 0.002198 |
|  | GO:0009719 | response to endogenous stimulus | 117/618 | 0.002303 |
|  | GO:0050789 | regulation of biological process | 220/1264 | 0.003897 |
|  | GO:0015979 | photosynthesis | 59/288 | 0.004983 |
|  | GO:0006950 | response to stress | 337/2023 | 0.006309 |
|  | GO:0040007 | growth | 74/381 | 0.007293 |
|  | GO:0009605 | response to external stimulus | 122/671 | 0.007470 |
|  | GO:0008152 | metabolic process | 1787/11823 | 0.026598 |
|  | GO:0009628 | response to abiotic stimulus | 171/1015 | 0.031411 |
|  | GO:0044710 | single-organism metabolic process | 225/1367 | 0.037115 |
|  |  | **CK2 versus A2** |  |  |
|  | GO:0015979 | photosynthesis | 90/288 | 0.000000 |
|  | GO:0006091 | generation of precursor metabolites and energy | 87/414 | 0.000000 |
|  | GO:0019748 | secondary metabolic process | 64/281 | 0.000000 |
|  | GO:0008152 | metabolic process | 1469/11823 | 0.000000 |
|  | GO:0009719 | response to endogenous stimulus | 114/618 | 0.000000 |
|  | GO:0006950 | response to stress | 291/2023 | 0.000026 |
|  | GO:0008219 | cell death | 41/186 | 0.000034 |
|  | GO:0009058 | biosynthetic process | 624/4776 | 0.000104 |
|  | GO:0050896 | response to stimulus | 423/3177 | 0.000481 |
|  | GO:0009607 | response to biotic stimulus | 82/504 | 0.000887 |
|  | GO:0009875 | pollen-pistil interaction | 23/107 | 0.002361 |
|  | GO:0007154 | cell communication | 177/1294 | 0.009140 |
|  | GO:0019725 | cellular homeostasis | 38/225 | 0.010875 |
|  | GO:0042592 | homeostatic process | 38/226 | 0.011655 |
|  | GO:0065008 | regulation of biological quality | 38/226 | 0.011655 |
|  | GO:0009605 | response to external stimulus | 96/671 | 0.016298 |
|  | GO:0007165 | signal transduction | 144/1076 | 0.033635 |
|  | GO:0050794 | regulation of cellular process | 144/1077 | 0.034522 |
|  | GO:0051716 | cellular response to stimulus | 144/1077 | 0.034522 |
|  | GO:0044710 | single-organism metabolic process | 179/1367 | 0.039609 |
|  | GO:0009628 | response to abiotic stimulus | 135/1015 | 0.045503 |
|  |  | **CK2 versus B2** |  |  |
|  | GO:0007154 | cell communication | 184/1294 | 0.000003 |
|  | GO:0006950 | response to stress | 268/2023 | 0.000005 |
|  | GO:0009875 | pollen-pistil interaction | 26/107 | 0.000025 |
|  | GO:0023052 | signaling | 149/1106 | 0.000343 |
|  | GO:0044700 | single organism signaling | 149/1106 | 0.000343 |
|  | GO:0007165 | signal transduction | 145/1076 | 0.000406 |
|  | GO:0050794 | regulation of cellular process | 145/1077 | 0.000423 |
|  | GO:0051716 | cellular response to stimulus | 145/1077 | 0.000423 |
|  | GO:0008219 | cell death | 34/186 | 0.000675 |
|  | GO:0009719 | response to endogenous stimulus | 89/618 | 0.000717 |
|  | GO:0009607 | response to biotic stimulus | 75/504 | 0.000725 |
|  | GO:0050896 | response to stimulus | 376/3177 | 0.001186 |
|  | GO:0019748 | secondary metabolic process | 43/281 | 0.005517 |
|  | GO:0009856 | pollination | 32/204 | 0.010695 |
|  | GO:0044703 | multi-organism reproductive process | 32/204 | 0.010695 |
|  | GO:0044706 | multi-multicellular organism process | 32/204 | 0.010695 |
|  | GO:0051704 | multi-organism process | 32/204 | 0.010695 |
|  | GO:0050789 | regulation of biological process | 154/1264 | 0.014754 |
|  | GO:0006464 | cellular protein modification process | 306/2658 | 0.016030 |
|  | GO:0036211 | protein modification process | 306/2658 | 0.016030 |
|  | GO:0043412 | macromolecule modification | 306/2658 | 0.016030 |
|  | GO:0044702 | single organism reproductive process | 73/555 | 0.017744 |
|  | GO:0008152 | metabolic process | 1255/11823 | 0.028814 |
|  | GO:0044763 | single-organism cellular process | 271/2388 | 0.041620 |
|  | GO:0065007 | biological regulation | 172/1474 | 0.042900 |
|  |  | **CK2 versus C2** |  |  |
|  | GO:0006950 | response to stress | 229/2023 | 0.000462 |
|  | GO:0009875 | pollen-pistil interaction | 21/107 | 0.000708 |
|  | GO:0009719 | response to endogenous stimulus | 80/618 | 0.001231 |
|  | GO:0009607 | response to biotic stimulus | 65/504 | 0.003682 |
|  | GO:0019748 | secondary metabolic process | 39/281 | 0.006721 |
|  | GO:0008152 | metabolic process | 1134/11823 | 0.009167 |
|  | GO:0009856 | pollination | 29/204 | 0.012955 |
|  | GO:0044703 | multi-organism reproductive process | 29/204 | 0.012955 |
|  | GO:0044706 | multi-multicellular organism process | 29/204 | 0.012955 |
|  | GO:0051704 | multi-organism process | 29/204 | 0.012955 |
|  | GO:0007154 | cell communication | 142/1294 | 0.015982 |
|  | GO:0008219 | cell death | 25/186 | 0.036616 |
|  |  | **CK2 versus D2** |  |  |
|  | GO:0015979 | photosynthesis | 95/288 | 0.000000 |
|  | GO:0009875 | pollen-pistil interaction | 42/107 | 0.000000 |
|  | GO:0006091 | generation of precursor metabolites and energy | 99/414 | 0.000014 |
|  | GO:0009856 | pollination | 56/204 | 0.000019 |
|  | GO:0044703 | multi-organism reproductive process | 56/204 | 0.000019 |
|  | GO:0044706 | multi-multicellular organism process | 56/204 | 0.000019 |
|  | GO:0051704 | multi-organism process | 56/204 | 0.000019 |
|  | GO:0009607 | response to biotic stimulus | 115/504 | 0.000028 |
|  | GO:0007154 | cell communication | 257/1294 | 0.000058 |
|  | GO:0009605 | response to external stimulus | 140/671 | 0.000361 |
|  | GO:0008152 | metabolic process | 1958/11823 | 0.000509 |
|  | GO:0006950 | response to stress | 368/2023 | 0.002132 |
|  | GO:0050896 | response to stimulus | 560/3177 | 0.002510 |
|  | GO:0019748 | secondary metabolic process | 63/281 | 0.002634 |
|  | GO:0008219 | cell death | 44/186 | 0.003902 |
|  | GO:0044702 | single organism reproductive process | 112/555 | 0.004196 |
|  | GO:0006464 | cellular protein modification process | 470/2658 | 0.004692 |
|  | GO:0036211 | protein modification process | 470/2658 | 0.004692 |
|  | GO:0043412 | macromolecule modification | 470/2658 | 0.004692 |
|  | GO:0044699 | single-organism process | 728/4231 | 0.005680 |
|  | GO:0022414 | reproductive process | 125/643 | 0.009213 |
|  | GO:0044763 | single-organism cellular process | 417/2388 | 0.016756 |
|  | GO:0040007 | growth | 76/381 | 0.020816 |
|  | GO:0044710 | single-organism metabolic process | 245/1367 | 0.022030 |
|  | GO:0009628 | response to abiotic stimulus | 185/1015 | 0.024352 |
|  | GO:0032501 | multicellular organismal process | 315/1795 | 0.028539 |
|  | GO:0009719 | response to endogenous stimulus | 116/618 | 0.031129 |
|  | GO:0009991 | response to extracellular stimulus | 23/99 | 0.037085 |
|  | GO:0009653 | anatomical structure morphogenesis | 144/788 | 0.039902 |
|  | GO:0007165 | signal transduction | 192/1076 | 0.045349 |
|  | GO:0050794 | regulation of cellular process | 192/1077 | 0.046706 |
|  | GO:0051716 | cellular response to stimulus | 192/1077 | 0.046706 |
| **Cellular Component** |  | **CK1 versus A1** |  |  |
|  | GO:0030312 | external encapsulating structure | 32/254 | 0.000000 |
|  | GO:0005618 | cell wall | 31/251 | 0.000000 |
|  | GO:0005576 | extracellular region | 34/414 | 0.000186 |
|  | GO:0071944 | cell periphery | 57/943 | 0.004287 |
|  | GO:0016020 | membrane | 175/3701 | 0.045149 |
|  | GO:0009579 | thylakoid | 23/372 | 0.048413 |
|  |  | **CK1 versus B1** |  |  |
|  | GO:0030312 | external encapsulating structure | 52/254 | 0.000000 |
|  | GO:0005618 | cell wall | 51/251 | 0.000000 |
|  | GO:0071944 | cell periphery | 121/943 | 0.000000 |
|  | GO:0016020 | membrane | 355/3701 | 0.000228 |
|  | GO:0009579 | thylakoid | 49/372 | 0.000850 |
|  | GO:0005576 | extracellular region | 52/414 | 0.001758 |
|  | GO:0005886 | plasma membrane | 78/728 | 0.011604 |
|  |  | **CK1 versus C1** |  |  |
|  | GO:0030312 | external encapsulating structure | 38/254 | 0.000000 |
|  | GO:0005618 | cell wall | 37/251 | 0.000000 |
|  | GO:0071944 | cell periphery | 81/943 | 0.000056 |
|  | GO:0016020 | membrane | 241/3701 | 0.001542 |
|  | GO:0005576 | extracellular region | 35/414 | 0.010191 |
|  | GO:0009579 | thylakoid | 32/372 | 0.010802 |
|  |  | **CK1 versus D1** |  |  |
|  | GO:0030312 | external encapsulating structure | 59/254 | 0.000007 |
|  | GO:0005618 | cell wall | 58/251 | 0.000010 |
|  | GO:0016020 | membrane | 553/3701 | 0.000025 |
|  | GO:0071944 | cell periphery | 164/943 | 0.000059 |
|  | GO:0009579 | thylakoid | 68/372 | 0.002775 |
|  | GO:0005886 | plasma membrane | 112/728 | 0.040670 |
|  |  | **CK2 versus A2** |  |  |
|  | GO:0009579 | thylakoid | 108/372 | 0.000000 |
|  | GO:0009536 | plastid | 212/1283 | 0.000000 |
|  | GO:0030312 | external encapsulating structure | 54/254 | 0.000002 |
|  | GO:0005618 | cell wall | 52/251 | 0.000007 |
|  | GO:0016020 | membrane | 477/3701 | 0.000012 |
|  | GO:0071944 | cell periphery | 135/943 | 0.001057 |
|  |  | **CK2 versus B2** |  |  |
|  | GO:0030312 | external encapsulating structure | 55/254 | 0.000000 |
|  | GO:0005618 | cell wall | 54/251 | 0.000000 |
|  | GO:0071944 | cell periphery | 139/943 | 0.000000 |
|  | GO:0016020 | membrane | 392/3701 | 0.000201 |
|  | GO:0005886 | plasma membrane | 93/728 | 0.000703 |
|  | GO:0005576 | extracellular region | 56/414 | 0.002371 |
|  |  | **CK2 versus C2** |  |  |
|  | GO:0030312 | external encapsulating structure | 44/254 | 0.000003 |
|  | GO:0005618 | cell wall | 43/251 | 0.000005 |
|  | GO:0016020 | membrane | 358/3701 | 0.000234 |
|  | GO:0071944 | cell periphery | 107/943 | 0.000627 |
|  | GO:0005576 | extracellular region | 46/414 | 0.030962 |
|  |  | **CK2 versus D2** |  |  |
|  | GO:0009579 | thylakoid | 109/372 | 0.000000 |
|  | GO:0030312 | external encapsulating structure | 70/254 | 0.000001 |
|  | GO:0016020 | membrane | 660/3701 | 0.000001 |
|  | GO:0071944 | cell periphery | 200/943 | 0.000001 |
|  | GO:0005618 | cell wall | 67/251 | 0.000003 |
|  | GO:0009536 | plastid | 248/1283 | 0.000059 |
|  | GO:0005886 | plasma membrane | 144/728 | 0.000939 |
| **Molecular_function** |  | **CK1 versus A1** |  |  |
|  | GO:0016740 | transferase activity | 236/3681 | 0.000000 |
|  | GO:0003700 | transcription factor activity, sequence-specific DNA binding | 60/823 | 0.000382 |
|  | GO:0001071 | nucleic acid binding transcription factor activity | 60/824 | 0.000394 |
|  | GO:0003824 | catalytic activity | 494/9718 | 0.001632 |
|  | GO:0016301 | kinase activity | 92/1625 | 0.027505 |
|  | GO:0016772 | transferase activity, transferring phosphorus-containing groups | 92/1627 | 0.028265 |
|  |  | **CK1 versus B1** |  |  |
|  | GO:0003700 | transcription factor activity, sequence-specific DNA binding | 131/823 | 0.000000 |
|  | GO:0001071 | nucleic acid binding transcription factor activity | 131/824 | 0.000000 |
|  | GO:0016740 | transferase activity | 386/3681 | 0.000017 |
|  | GO:0016301 | kinase activity | 187/1625 | 0.000034 |
|  | GO:0016772 | transferase activity, transferring phosphorus-containing groups | 187/1627 | 0.000037 |
|  | GO:0003677 | DNA binding | 175/1649 | 0.003023 |
|  | GO:0019825 | oxygen binding | 8/39 | 0.017581 |
|  | GO:0005488 | binding | 945/10382 | 0.017886 |
|  | GO:0005102 | receptor binding | 3/9 | 0.037288 |
|  |  | **CK1 versus C1** |  |  |
|  | GO:0003700 | transcription factor activity, sequence-specific DNA binding | 90/823 | 0.000000 |
|  | GO:0001071 | nucleic acid binding transcription factor activity | 90/824 | 0.000000 |
|  | GO:0005102 | receptor binding | 3/9 | 0.012907 |
|  | GO:0003677 | DNA binding | 116/1649 | 0.020805 |
|  |  | **CK1 versus D1** |  |  |
|  | GO:0003700 | transcription factor activity, sequence-specific DNA binding | 199/823 | 0.000000 |
|  | GO:0001071 | nucleic acid binding transcription factor activity | 199/824 | 0.000000 |
|  | GO:0016740 | transferase activity | 601/3681 | 0.000575 |
|  | GO:0003677 | DNA binding | 281/1649 | 0.002195 |
|  | GO:0005102 | receptor binding | 5/9 | 0.004994 |
|  | GO:0003824 | catalytic activity | 1477/9718 | 0.007853 |
|  | GO:0016301 | kinase activity | 267/1625 | 0.017047 |
|  | GO:0016772 | transferase activity, transferring phosphorus-containing groups | 267/1627 | 0.018000 |
|  | GO:0005488 | binding | 1565/10382 | 0.020075 |
|  | GO:0003674 | molecular_function | 2607/17817 | 0.031619 |
|  |  | **CK2 versus A2** |  |  |
|  | GO:0003700 | transcription factor activity, sequence-specific DNA binding | 153/823 | 0.000000 |
|  | GO:0001071 | nucleic acid binding transcription factor activity | 153/824 | 0.000000 |
|  | GO:0016740 | transferase activity | 507/3681 | 0.000000 |
|  | GO:0003824 | catalytic activity | 1181/9718 | 0.000128 |
|  | GO:0003677 | DNA binding | 226/1649 | 0.001150 |
|  | GO:0016772 | transferase activity, transferring phosphorus-containing groups | 212/1627 | 0.015304 |
|  | GO:0016301 | kinase activity | 211/1625 | 0.017774 |
|  | GO:0030234 | enzyme regulator activity | 32/202 | 0.032341 |
|  | GO:0098772 | molecular function regulator | 32/202 | 0.032341 |
|  |  | **CK2 versus B2** |  |  |
|  | GO:0016740 | transferase activity | 524/3681 | 0.000000 |
|  | GO:0003700 | transcription factor activity, sequence-specific DNA binding | 135/823 | 0.000000 |
|  | GO:0001071 | nucleic acid binding transcription factor activity | 135/824 | 0.000000 |
|  | GO:0003824 | catalytic activity | 1102/9718 | 0.000000 |
|  | GO:0016772 | transferase activity, transferring phosphorus-containing groups | 226/1627 | 0.000000 |
|  | GO:0016301 | kinase activity | 225/1625 | 0.000001 |
|  | GO:0036094 | small molecule binding | 459/3911 | 0.000201 |
|  | GO:0000166 | nucleotide binding | 458/3904 | 0.000213 |
|  | GO:1901265 | nucleoside phosphate binding | 458/3904 | 0.000213 |
|  | GO:0005102 | receptor binding | 5/9 | 0.000968 |
|  | GO:0030246 | carbohydrate binding | 38/239 | 0.003758 |
|  | GO:0030234 | enzyme regulator activity | 29/202 | 0.036697 |
|  | GO:0098772 | molecular function regulator | 29/202 | 0.036697 |
|  |  | **CK2 versus C2** |  |  |
|  | GO:0016740 | transferase activity | 454/3681 | 0.000000 |
|  | GO:0003700 | transcription factor activity, sequence-specific DNA binding | 126/823 | 0.000000 |
|  | GO:0001071 | nucleic acid binding transcription factor activity | 126/824 | 0.000000 |
|  | GO:0016301 | kinase activity | 197/1625 | 0.000027 |
|  | GO:0016772 | transferase activity, transferring phosphorus-containing groups | 197/1627 | 0.000030 |
|  | GO:0003824 | catalytic activity | 969/9718 | 0.000102 |
|  | GO:0003677 | DNA binding | 183/1649 | 0.004079 |
|  | GO:0005102 | receptor binding | 4/9 | 0.006238 |
|  | GO:0030234 | enzyme regulator activity | 28/202 | 0.019395 |
|  | GO:0098772 | molecular function regulator | 28/202 | 0.019395 |
|  | GO:0030246 | carbohydrate binding | 31/239 | 0.033400 |
|  | GO:0000166 | nucleotide binding | 388/3904 | 0.045946 |
|  | GO:1901265 | nucleoside phosphate binding | 388/3904 | 0.045946 |
|  |  | **CK2 versus D2** |  |  |
|  | GO:0016740 | transferase activity | 723/3681 | 0.000000 |
|  | GO:0003824 | catalytic activity | 1667/9718 | 0.000000 |
|  | GO:0016772 | transferase activity, transferring phosphorus-containing groups | 338/1627 | 0.000000 |
|  | GO:0016301 | kinase activity | 337/1625 | 0.000000 |
|  | GO:0005102 | receptor binding | 8/9 | 0.000003 |
|  | GO:0003700 | transcription factor activity, sequence-specific DNA binding | 166/823 | 0.000178 |
|  | GO:0001071 | nucleic acid binding transcription factor activity | 166/824 | 0.000189 |
|  | GO:0000166 | nucleotide binding | 654/3904 | 0.011240 |
|  | GO:1901265 | nucleoside phosphate binding | 654/3904 | 0.011240 |
|  | GO:0036094 | small molecule binding | 655/3911 | 0.011413 |
|  | GO:0030246 | carbohydrate binding | 50/239 | 0.016021 |
