# Supplementary figures and images for: Synthesis of light-inducible and light-independent anthocyanins regulated by specific genes in grape ‘Marselan’ (V. vinifera L.)
Source: PeerJ. 2019 Mar 1;7:e6521. doi: 10.7717/peerj.6521 (PMC6398381; doi:10.7717/peerj.6521)

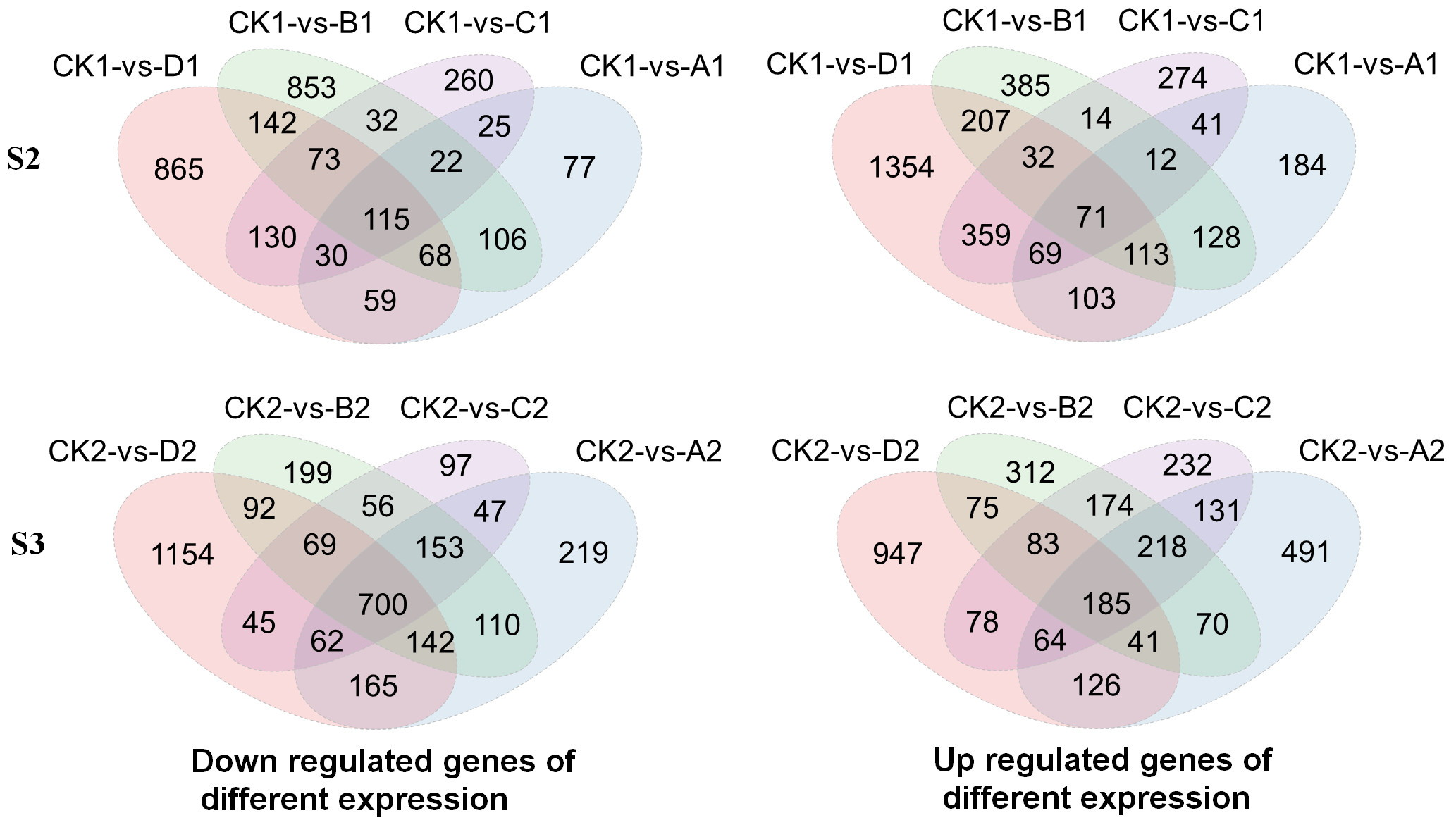

Supplement: Supplemental Information 3 [file peerj-07-6521-s003.png]
